# Effect of omega-3 polyunsaturated fatty acids supplementation for patients with osteoarthritis: a meta-analysis

**DOI:** 10.1186/s13018-023-03855-w

**Published:** 2023-05-24

**Authors:** Wen Deng, Zhiqian Yi, Enzhi Yin, Rui Lu, Hongbo You, Xuefeng Yuan

**Affiliations:** 1 Medical Affairs Office, Tongji Hospital, Tongji Medical College, Huazhong University of Science and Technology, Wuhan, 430030 China; 2 Department of Orthopedics, Tongji Hospital, Tongji Medical College, Huazhong University of Science and Technology, Wuhan, 430030 China; 3 Department of Traumatology, Tongji Hospital, Tongji Medical College, Huazhong University of Science and Technology, Jie Fang Avenue 1095, Wuhan, 430030 China

**Keywords:** Osteoarthritis, Omega-3 polyunsaturated fatty acids, Arthritis pain, Joint function, Meta-analysis

## Abstract

**Background:**

Omega-3 polyunsaturated fatty acids (n-3 PUFAs) confers anti-inflammatory efficacy, which has been suggested to be effective for patients with osteoarthritis (OA). However, previous studies evaluating the influence of n-3 PUFAs supplementation in patients with OA showed inconsistent results. We performed a systematic review and meta-analysis to comprehensively evaluate the influence of n-3 PUFAs on symptom and joint function of patients with OA.

**Methods:**

Relevant randomized controlled trials (RCTs) were obtained by searching PubMed, Embase, and Cochrane Library databases. A random-effects model was employed to combine the results.

**Results:**

Nine RCTs with 2070 patients with OA contributed to the meta-analysis. Pooled results showed that n-3 PUFAs supplementation could significantly relieve the arthritis pain as compared to placebo (standardized mean difference [SMD]: − 0.29, 95% confidence interval [CI] − 0.47 to − 0.11, *p* = 0.002, *I*^2^ = 60%). Besides, supplementation with n-3 PUFAs was also associated with improved joint function (SMD: − 0.21, 95% CI − 0.34 to − 0.07, *p* = 0.002, *I*^2^ = 27%). Subgroup analysis showed consistent results of studies with arthritis pain and joint function evaluated by the Western Ontario-McMaster University Osteoarthritis Index and other scales (*p* for subgroup difference = 0.33 and 0.34, respectively). No severe treatment-related adverse events (AEs) were observed in the included patients, and the incidence of overall AEs was similar between groups (odds ratio: 0.97, 95% CI 0.64–1.45, *p* = 0.86, *I*^2^ = 0%).

**Conclusions:**

Supplementation of n-3 PUFAs is effective to relieve pain and improve joint function in patients with OA.

## Introduction

Worldwide, osteoarthritis (OA) is the most common degenerative joint disease affecting cartilage and surrounding tissues, which has become a leading cause of disability worldwide, particularly of the older population [1, 2]. With a growing elderly and obese population, the incidence of OA in recent decades has been increasing, and this has also resulted in a substantial economic burden for the global populations [3, 4]. With the improved understanding of the pathogenesis of OA, a variety of medications have been used in these patients, such as analgesics and non-steroidal anti-inflammatory drugs (NSAIDs), methotrexate, hydroxylchloroquine, and tumor necrosis factor (TNF) inhibitors [5, 6]. However, due to the potential adverse events associated with the long-term use of these medications and the poor efficacies of these agents in some patients with OA, substantial patients have been seeking alternative and complementary agents for relieving the symptoms and improving the function of the affected arthritis [7, 8]. Omega-3 polyunsaturated fatty acids (n-3 PUFAs), mainly including eicosapentaenoic acid (EPA) and docosahexanoic acid (DHA) [9], have been suggested to be effective for patients with OA because of their efficacy for attenuating the systemic inflammatory response [10] and the catabolic environment that accelerates cartilage degradation [11]. However, previous randomized controlled trials (RCTs) evaluating the efficacy of n-3 PUFAs for patients with OA showed inconsistent results. Some studies suggested that additional supplementation of n-3 PUFAs for patients with OA could improve the arthritis pain [11–13], while the others did not [14–19]. Therefore, we performed a systematic review and meta-analysis to comprehensively evaluate the influence of n-3 PUFAs on symptom and joint function of patients with OA.

## Methods

This study was designed and implemented according to the PRISMA (Preferred Reporting Items for Systematic Reviews and Meta-Analyses) statement [20, 21] and Cochrane Handbook guidelines [22].

### Search strategy

The Medline (PubMed), Embase (Ovid), and CENTER (Cochrane Library), databases were searched for relevant studies with a combined strategy of: (1) “omega-3 fatty acids” OR “fish oil” OR fish-oil OR “polyunsaturated fatty acids” OR “marine oil” OR “eicosapentaenoic acid” OR “docosahexanoic acid” OR “DHA” OR “EPA”; (2) “osteoarthritis” OR “osteoarthritic”; and (3) “random” OR “randomly” OR “randomized” OR “randomized” OR “control” OR “placebo”. Relevant studies were limited to the studies that included human subjects. Moreover, references of related reviews and original articles were also searched. The final database searches were conducted on January 6, 2023.

### Study selection

Included were studies with the following criteria: (1) full-length English articles; (2) RCTs with parallel groups; (3) patients with OA were randomly assigned to an intervention group of PUFAs supplementation on the basis of conventional treatments, and a control group of conventional treatments only (with placebo or no additional treatment); (4) one or more of the following efficacy and/or safety outcomes were reported, including the changes of arthritis pain, joint function, and the incidence of treatment related adverse events (AEs). The assessment of arthritis pain and joint function was in accordance with the methods and scales of the original studies. Non-randomized studies, studies not with patients of OA, studies not with an intervention group of n-3 PUFA supplementation, studies not with a control group of placebo or no treatment, or studies that failed to report the outcome of interest were excluded. In cases of studies with overlapped patient populations, the study with the largest sample size was included in the meta-analysis.

### Data extraction and quality assessment

Data extraction, data mining, and quality evaluation were handled by two independent authors. If disagreements arose, discussions between the two authors were made to reach consensus. The following variables were extracted, such as the publication detail (first author, publication year, and study country), study design (blinded or open-label), patient characteristics (number of patients, mean age, and sex), intervention (daily supplemental dose of n-3 PUFAs, and the contents of EPA and DHA), regimen of controls, follow-up durations, and the outcomes that were reported. We evaluated the quality of the study using Cochrane's Risk of Bias Tool [22] in accordance with the following criteria: (1) randomly generation of sequences; (2) concealing allocations; (3) blinding of participants and staff; (4) blinding outcome assessors; (5) presenting incomplete outcome data; (6) reporting selective results; and (7) other potential bias.

### Statistical analysis

Differences for the changes of pain and joint function after treatment between the intervention and control groups were summarized as standardized mean difference (MD) with corresponding 95% confidence interval (CI), because the scales used among the included studies were varying [22]. Influences of treatment on the outcomes of categorized variables were presented as odds ratios (OR) and corresponding CI. For detection of heterogeneity, we used Cochrane Q test [23]. A statistical analysis of heterogeneity was also conducted by estimating the *I*^2^ statistic, and an *I*^2^ > 50% suggests significant heterogeneity [24]. A random-effects model was used in the pooled analyses to account for potential heterogeneity and provide a more general conclusion [22]. In case of significant heterogeneity, a univariate meta-regression analysis was performed to explore the potential source of heterogeneity [22]. Sensitivity analysis by excluding one study at a time was performed to evaluate the robustness of the finding [22]. Moreover, subgroup analyses were performed to evaluate the potential influences of study characteristics on the outcome, such as the scales used, mean ages and sex of the patients, daily supplemental doses of n-3 PUFAs, EPA and DHA, and follow-up durations. An analysis of funnel plots and Egger’s regression asymmetry test was conducted when at least ten studies were included in order to determine publication bias [25]. Statistically significant differences were defined as *p* < 0.05. RevMan (version 5.1; Cochrane, Oxford, UK) and Stata (version 12.0; Stata Corporation) software were used for the statistical analyses.

## Results

### Search results

**Fig. 1:**
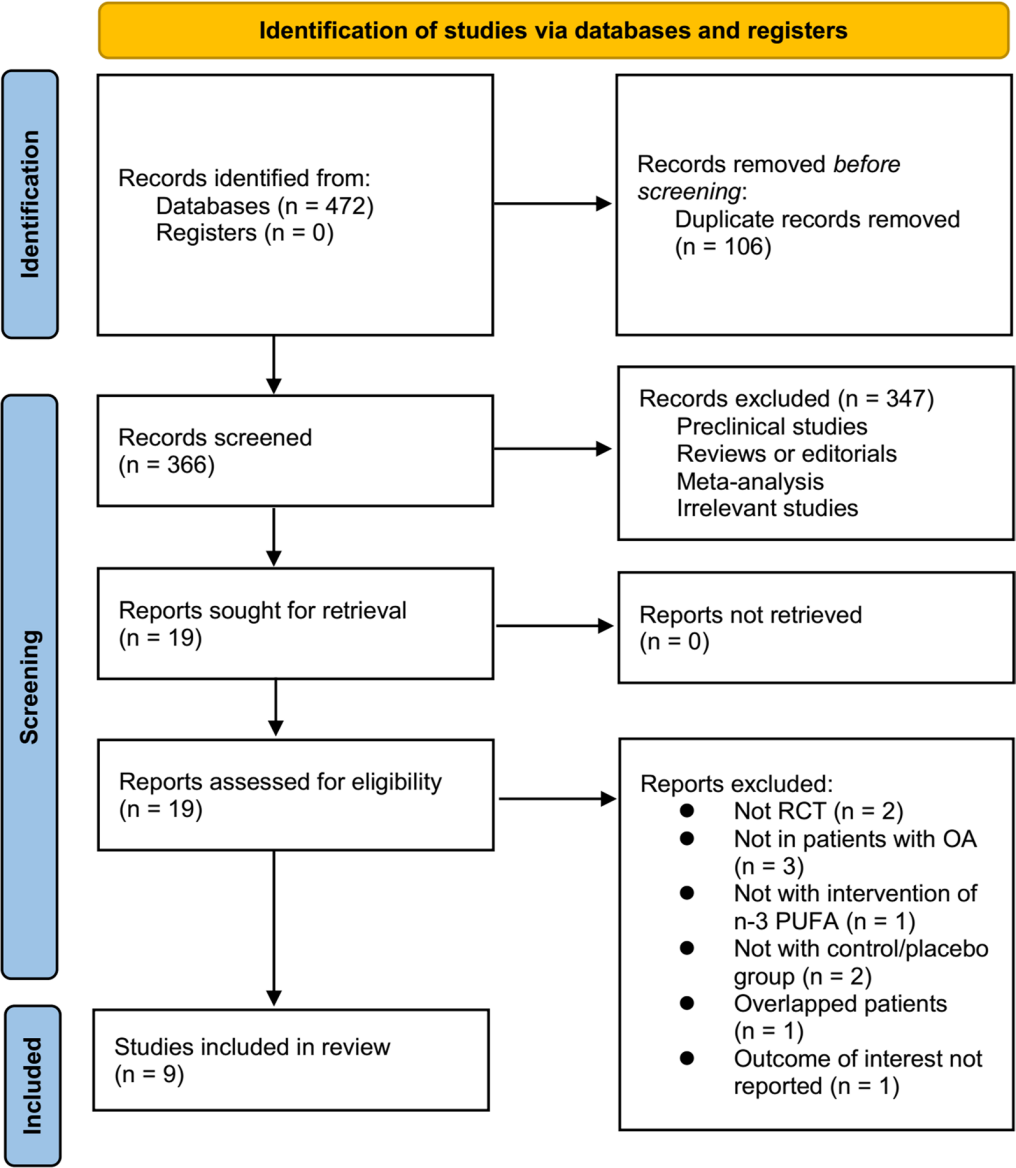
Flowchart of literature search

A diagram showing how to search databases and identify studies is shown in Fig. 1. By searching the database, 472 articles were obtained, and 366 were identified after excluding duplicates. Based on title and abstract, 347 of them were subsequently excluded, mainly because their objectives were irrelevant. Ten articles were further excluded from full-text review for the reasons illustrated in Fig. 1. The final analysis included nine RCTs [11–19] in total.

### Study characteristics and data quality

**Fig. 2:**
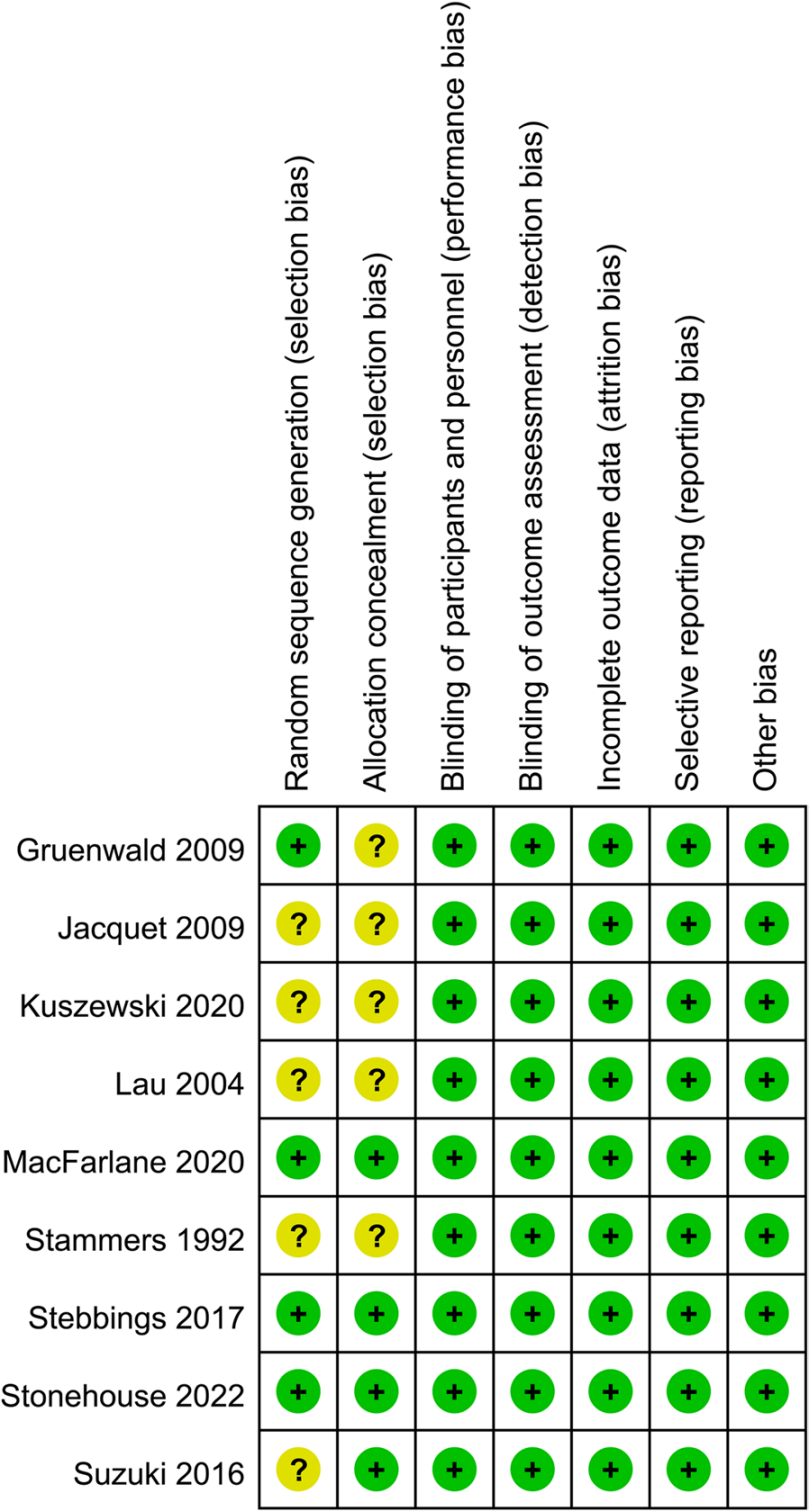
Summarized details of study quality evaluation via the Cochrane Risk of Bias Tool

An overview of the included studies is presented in Table 1**.** Overall, nine RCTs [11–19] including 2070 patients with OA contributed to the meta-analysis. These studies were all double-blind RCTs from the UK [14], China [11], France [12], Germany [15], Japan [16], New Zealand [17], the USA [19], and Australia [13, 18], which were published between 1992 and 2022. As for the diagnosis, three studies included patients with knee OA [13, 16, 19], four with hip or knee OA [11, 12, 15, 17], and the other two did not specify the sites of OA [14, 18]. The mean ages of the patients were 55 to 68 years, and the percentiles of men were 12–45%. A total of 1020 patients were allocated to the intervention group, who received a daily supplementation of n-3 PUFA of 350–2400 mg, and a total of 1050 patients were allocated to the control group of placebo or no additional treatment. The follow-up durations were 1 to 63 months. The arthritis pain was evaluated via visual analogue scale (VAS) in four studies [11, 14, 16, 18], and via Western Ontario-McMaster University Osteoarthritis Index (WOMAC) in five studies [12, 13, 15, 17, 19]. The joint function was evaluated via WOMAC in five studies [12, 13, 15, 17, 19], and with VAS [14], the Patients’ Global Assessment [11], and the Japanese Orthopaedic Association score [16] in the other three studies. Details of quality evaluation via the Cochrane Risk of Bias Tool for each included study are summarized in Fig. 2. All of the included studies were double-blind RCTs. Details of random sequence generation were reported in four studies [13, 15, 17, 19], and details of allocation concealment were also reported in four studies [13, 16, 17, 19]. No other publication biases such are those related to incomplete outcome data, selective reporting, or of other sources were detected.

**Table 1:** Characteristics of the included studies

Study	Country	Study design	OA site	Sample size	Mean age	Men	n-3 PUFA dose	EPA dose	DHA dose	Control	Follow-up duration	Outcomes reported
					years	%	mg/d	mg/d	mg/d		months	
Stammers 1992	UK	R, DB, PC	Not specified	86	68	27.9	786	786	0	Olive oil	6	VAS for pain and VAS for function
Lau 2004	China	R, DB, PC	Hip or knee	80	62.5	13.8	1360	520	840	Olive oil	6	VAS for pain and PGA for function
Jacquet 2009	France	R, DB, PC	Hip or knee	81	57.1	32.1	1360	520	840	Placebo	3	WOMAC for pain and function
Gruenwald 2009	Germany	R, DB	Hip or knee	177	62.3	36.5	1332	800	532	Palm and sunflower oil	6	WOMAC for pain and function
Suzuki 2016	Japan	R, DB, PC	Knee	47	64.7	12.8	350	240	110	Safflower oil	1	VAS for pain and JOA for function
Stebbings 2017	New Zealand	R, DB, PC	Hip or knee	80	66.4	45	460	280	180	Corn oil	4	WOMAC for pain and function
MacFarlane 2020	USA	R, DB, PC	Knee	1221	67.7	34	840	490	350	Placebo	63	WOMAC for pain and function
Kuszewski 2020	Australia	R, DB, PC	Not specified	63	65.4	NR	2400	400	2000	Corn oil	4	VAS for pain
Stonehouse 2022	Australia	R, DB, PC	Knee	235	55.9	45.1	880	600	280	Vegetable oil	6	WOMAC for pain and function

*n-3 PUFA* omega-3 polyunsaturated fatty acids; *OA* osteoarthritis; *EPA* eicosapentaenoic acid; *DHA* docosahexanoic acid; *R* randomized; *DB* double-blind; *PC* placebo-controlled; *VAS* visual analogue scale; *PGA* Patients’ Global Assessment; *WOMAC* Western Ontario-McMaster University Osteoarthritis Index; *JOA* Japanese Orthopaedic Association score

### Meta-analysis of n-3 PUFA supplementation on arthritis pain in patients with OA

Meta-analysis with nine studies evaluated the influences of n-3 PUFA supplementation on arthritis pain in patients with OA [11–19]. Significant heterogeneity was observed among these studies (*p* for Cochrane Q test = 0.01, *I*^2^ = 60%). Pooled results showed that n-3 PUFAs supplementation could significantly relieve the arthritis pain as compared to placebo (SMD: − 0.29, 95% CI − 0.47 to − 0.11, *p* = 0.002; Fig. 2A). Results of univariate meta-regression analysis failed to show the following variables may significantly contribute to the heterogeneity, including patient number, mean age, percentile of men, dose of n-3 PUFA, EPA, or DHA, and the follow-up duration (Table 2, *p* all > 0.05). Sensitivity analysis by excluding one study at a time showed consistent results (− 0.22 to − 0.34, p all < 0.05). Subgroup analysis showed that the improvement of pain after n-3 PUFA supplementation was consistent in studies with the symptom evaluated by WOMAC or VAS, in studies with n-3 PUFA supplementation < 1000 mg/d or ≥ 1000 mg/d, with EPA < 500 mg/d or ≥ 500 mg/d, with DHA < 500 mg/d or ≥ 500 mg/d, or with follow-up duration < 6 or ≥ 6 months (p for subgroup difference all > 0.05; Table 3). Interestingly, a more remarkable improvement in arthritis pain was observed in younger patients (< 65 years) than it in older patients (≥ 65 years, p for subgroup difference = 0.03; Table 3).

**Table 2:** Univariate meta-regression analysis for the effects of n-3 PUFA on joint pain in patients with OA

Covariate	Pain		
	Coefficient	95% CI	*p*
Patient number	0.0003	− 0.0001 to 0.0007	0.16
Mean age (years)	0.031	− 0.012 to 0.073	0.13
Men (%)	0.0092	− 0.0117 to 0.0301	0.33
n-3 PUFA dose (mg/d)	− 0.0002	− 0.0006 to 0.0002	0.21
EPA dose (mg/d)	0.0001	− 0.0012 to 0.0014	0.82
DHA dose (mg/d)	− 0.0003	− 0.0007 to 0.0001	0.18
Follow-up duration (months)	0.005	− 0.003 to 0.013	0.18

*CI* confidence interval; *n-3 PUFA* omega-3 polyunsaturated fatty acids; *OA* osteoarthritis; *EPA* eicosapentaenoic acid; *DHA* docosahexanoic acid

**Table 3:** Subgroup analysis

	Arthritis pain of patients with OA					Joint function of patients with OA				
Study characteristics	Datasets number	SMD (95% CI)	*I*^2^ (%)	P1	P2	Datasets number	SMD (95% CI)	*I*^2^ (%)	P1	P2
*Scale*										
WOMAC	5	− 0.23 [− 0.45, − 0.02]	67	0.03		5	− 0.19 [− 0.36, − 0.02]	49	0.03	
Others	4	− 0.40 [− 0.67, − 0.13]	19	0.003	0.33	3	− 0.35 [− 0.65, − 0.05]	0	0.02	0.34
*Mean age*										
< 65 years	5	− 0.40 [− 0.67, − 0.13]	60	0.004		5	− 0.29 [− 0.50, − 0.08]	31	0.006	
≥ 65 years	4	− 0.09 [− 0.19, 0.02]	0	0.10	0.03	3	− 0.12 [− 0.23, − 0.01]	0	0.03	0.15
*Dose of n*− *3 PUFA*										
< 1000 mg/d	5	− 0.11 [− 0.21, − 0.01]	0	0.02		5	− 0.14 [− 0.24, − 0.05]	0	0.004	
≥ 1000 mg/d	4	− 0.49 [− 0.86, − 0.11]	69	0.01	0.09	3	− 0.37 [− 0.80, 0.06]	65	0.09	0.31
*Dose of EPA*										
< 500 mg/d	4	− 0.08 [− 0.19, 0.02]	0	0.12		3	− 0.11 [− 0.22, − 0.00]	0	0.04	
≥ 500 mg/d	5	− 0.39 [− 0.65, − 0.14]	60	0.003	0.08	5	− 0.31 [− 0.51, − 0.10]	33	0.003	0.09
*Dose of DHA (mg/d)*										
< 500 mg/d	5	− 0.11 [− 0.21, − 0.01]	0	0.02		5	− 0.14 [− 0.24, − 0.05]	0	0.004	
≥ 500 mg/d	4	− 0.49 [− 0.86, − 0.11]	69	0.01	0.09	3	− 0.37 [− 0.80, 0.06]	65	0.09	0.31
*Follow− up duration*										
< 6 months	4	− 0.39 [− 0.70, − 0.09]	38	0.01		3	− 0.33 [− 0.77, 0.11]	60	0.15	
≥ 6 months	5	− 0.23 [− 0.44, − 0.03]	65	0.03	0.39	5	− 0.14 [− 0.24, − 0.05]	0	0.002	0.42

*P1* *p* values for subgroup effect; *P2* *p* values for subgroup difference; *OA* osteoarthritis; *SMD* standardized mean difference; *CI* confidence interval; *n-3 PUFA* omega-3 polyunsaturated fatty acids; *EPA* eicosapentaenoic acid; *DHA* docosahexanoic acid; *WOMAC* Western Ontario-McMaster University Osteoarthritis Index

### Meta-analysis of n-3 PUFA supplementation on joint function in patients with OA

Pooled results of eight studies [11–17, 19] showed a significant improved joint function following n-3 PUFA supplementation in patients with OA (SMD: − 0.21, 95% CI: − 0.34 to − 0.07, *p* = 0.002; Fig. 2B) with mild heterogeneity (*p* for Cochrane Q test = 0.21, *I*^2^ = 27%). Sensitivity analysis by omitting one study at a time did not significantly affect the results (− 0.22 to − 0.34, *p* all < 0.05). In addition, subgroup analysis showed consistent results of studies with joint function evaluated by WOMAC or other scales (*p* for subgroup difference = 0.34, Table 3). Moreover, further subgroup analysis according to the mean age of the patients, daily dose of n-3 PUFA, EPA, and DHA, as well as the follow-up durations also showed consistent results (*p* for subgroup difference all > 0.05; Table 3).

### Safety outcome

No severe AEs related to the treatment n-3 PUFA were reported among the included studies. Pooled results with seven studies [11–15, 17, 18] showed that the incidence of treatment-related AEs was not significantly different between groups (OR: 0.97, 95% CI 0.64–1.45, *p* = 0.86, *I*^2^ = 0%; Fig. 2C).

### Publication bias

**Fig. 3:**
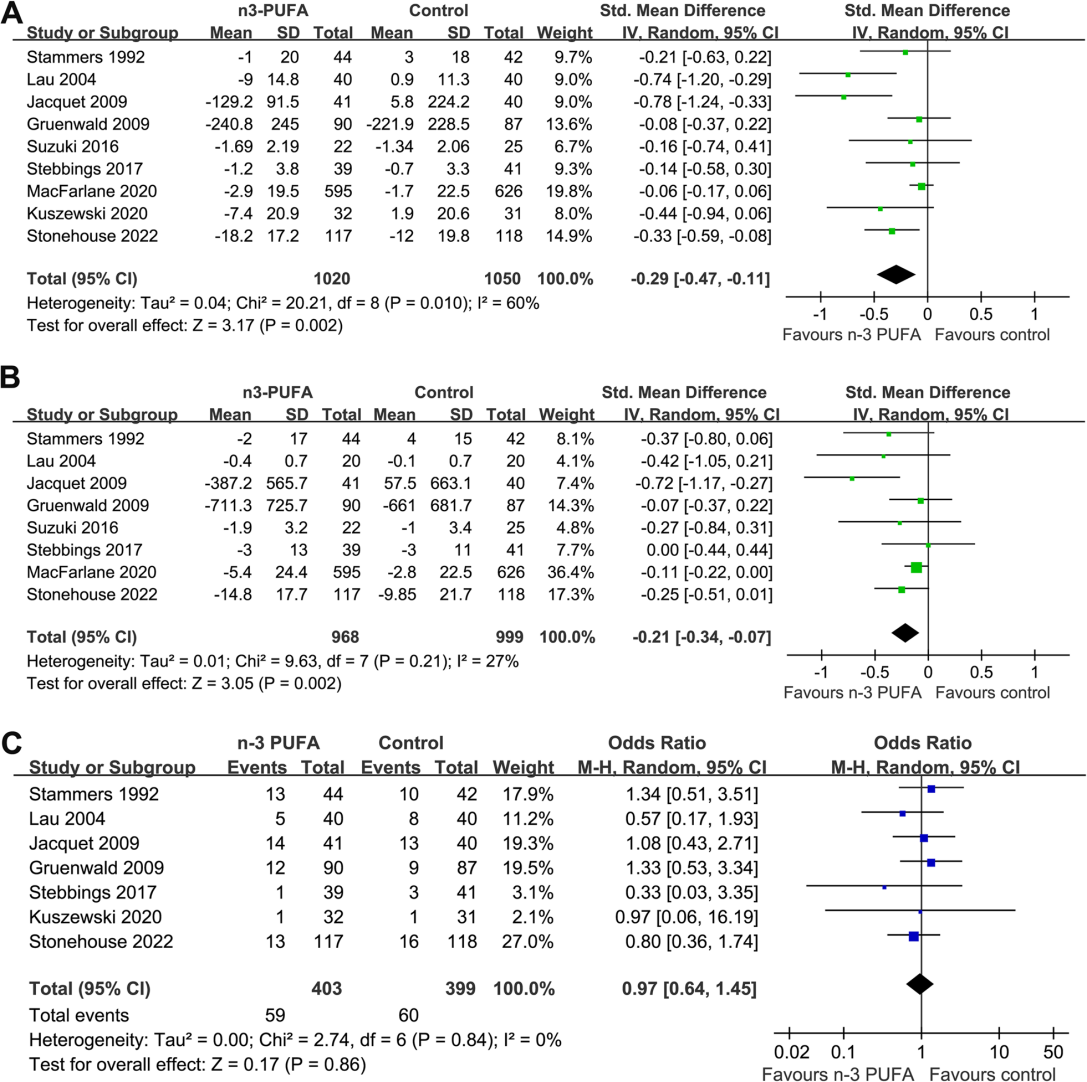
Forest plots for the meta-analysis of the efficacy and safety of n-3 PUFA supplementation in patients with OA; **A**, Forest plots for the meta-analysis of arthritis pain; **B**, Forest plots for the meta-analysis of joint function; and **C**, Forest plots for the meta-analysis of the incidence of treatment related AEs

**Fig. 4:**
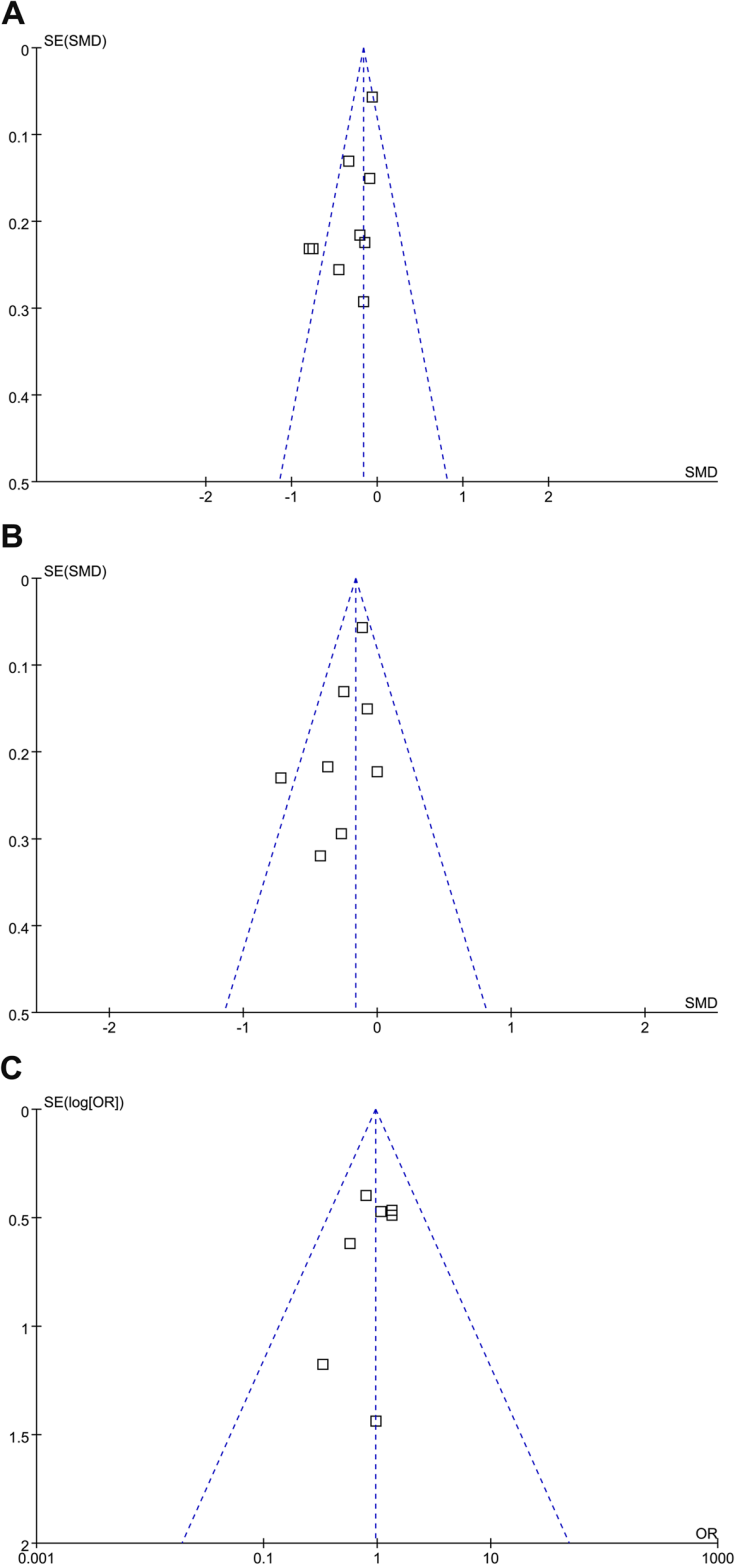
Funnel plots for the detection of the publication biases underlying the meta-analysis; **A**, Funnel plots for the meta-analysis of arthritis pain; **B**, Funnel plots for the meta-analysis of joint function; and **C**, Funnel plots for the meta-analysis of the incidence of treatment related AEs

The funnel plots for the meta-analyses of the influences of n-3 PUFA supplementation on arthritis pain, joint function, and the incidence of AEs are shown in Fig. 3A, B, C. These plots were symmetrical on visual inspection, reflecting low risks of publication biases. In addition, results of Egger’s regression tests also confirmed the low risks of publication biases (*p* = 0.26, 0.31, and 0.19, respectively) (Fig. 4).

## Discussion

In this systematic review and meta-analysis, we pooled the results of nine relevant RCTs, and the results showed that compared to placebo or no additional treatment, supplementation of n-3 PUFA could significantly relieve arthritis pain and improve joint function in patients with OA. Moreover, no severer treatment related AEs were reported, and the incidence of overall treatment related AEs was comparable between n-3 PUFA and controls. Taking together, these results suggest that supplementation of n-3 PUFAs is effective and safe in patients with OA.

To the best of our knowledge, few meta-analyses have been published which evaluated the role of n-3 PUFAs supplementation as an alternative treatment for patients with OA. An early meta-analysis by Senftleber et al. in 2017 evaluated the influences of marine oil supplements for arthritis pain of various etiologies [26]. Although they found that supplementation of marine oil may be effective to relieve arthritis pain, most of the studies included patients with rheumatoid arthritis, and a subgroup analysis of five studies of patients with OA failed to show the potential efficacy of marine oil on arthritis pain [26]. Compared to the previous meta-analysis, our study has some potential methodological strength. First, extensive literature search was performed in three commonly used electronic databases, which could provide the up-to-date studies regarding the role of n-3 PUFA supplementation in patients with OA. Second, only RCTs were included, and all of the included studies were of double-blind design, which could minimize the influences of patient characteristics on the outcomes of the meta-analysis. Third, besides the symptom of pain, influences of n-3 PUFA supplementation on joint function was also evaluated, as well as the safety outcome. Finally, besides the main meta-analysis, a few sensitivity and subgroup analyses were performed to evaluate the robustness of the findings. The generally consistent results of these analyses further confirmed the stability of the results.

Overall, we found that n-3 PUFA is effective in relieving pain and improving joint function in patients with OA. The mechanisms underlying the benefits of n-3 PUFA may be multifactorial. A previous study in a cellular model for canine OA suggested that EPA and DHA supplementation could reduce the expression of multiple inflammatory markers involved in the pathogenesis of cartilage degeneration, such as interleukin-1 beta (IL-1β) and inducible nitric oxide synthase [27]. A similar mechanism has also been revealed in leptin-induced cartilage degeneration, which showed that supplementation with EPA and DHA could reduce IL-1β-induced activation of nuclear factor-κB and c-Jun N-terminal kinase, thereby attenuating leptin-induced cartilage degeneration [28]. Besides, EPA has also been reported to reduce oxidative stress-induced apoptosis and matrix loss of chondrocytes by inhibiting metalloproteinases13 expression and chondrocyte apoptosis [29]. In addition, a previous study also showed that in a rat model of anterior cruciate ligament transection induced OA, DHA is effective to restrain bone remodeling and vessel formation in the osteochondral unit [30]. Furthermore, a recent study showed that maresin-1, a metabolite of DHA, also confers the therapeutic efficacy for OA in a rat model via its potential anti-inflammatory effects [31]. Studies are warranted to determine the key molecular pathways underlying the potential therapeutic efficacy of n-3 PUFA for OA.

Significant heterogeneity was observed for the meta-analysis of the influence of n-3 PUFA supplementation on arthritis pain in patients with OA. However, subsequent meta-regression and subgroup analysis failed to show that the heterogeneity could be explained by the predefined study characteristics, such as sample size, patient age, dose of n-3 PUFA, DHA, EPA, follow-up duration, or the scales for assessment of pain. From our perspective, the serum level of n-3 PUFA of the patients may be an important determinant for the effects of n-3 PUFA supplementation for OA. For patients with adequate dietary intake of n-3 PUFA (such as patients with habitual intake of fish), additional supplementation is unnecessary and of limited efficacy [32]. Unfortunately, none of the included studies reported the baseline level of n-3 PUFA in these patients. Further studies are warranted our hypothesis. Interestingly, for the outcome of arthritis pain, results of the meta-analysis suggested that benefits of n-3 PUFA supplementation may be more remarkable in younger patients (< 65 years) as compared to older patients (≥ 65 years). This may be explained that in older adults, OA is likely to frequently exist alongside other common chronic conditions which may deteriorate the symptom and function of the joint, such as diabetes [33], which limited the efficacy of n-3 PUFA supplementation. Similarly, studies are warranted in the future for further validation. Additionally, subgroup analyses failed to show more remarkable benefits of high-dose versus low-dose n-3 PUFA in patients with OA, which is also consistent with the finding of a previous clinical study [34].

This meta-analysis also has several limitations. First, the number of included study is limited. The results of the meta-analysis should be validated in large-scale RCTs, and the results of the subgroup analyses should be interpreted with caution. In addition, the optimal dose, components (ratio of EPA to DHA), and treatment duration of n-3 PUFA supplementation for OA remains to be determine. Furthermore, differences of n-3 PUFA doses and follow-up durations in different studies may affect the outcome of the meta-analysis, which may be a limitation of the current meta-analysis. In view of the fact that these variations may lead to clinical heterogeneity among the included studies, a random-effects model was used to pool the results. Besides, we performed meta-regression and subgroup analyses to evaluate if these factors may have significant influence on the outcome. However, these subsequent meta-regression and subgroup analyses failed to show that difference of these factors have a significant influence on the results of the meta-analysis. Moreover, as mentioned previously, influences of dietary habits and concomitant treatment on the efficacy of n-3 PUFA supplementation for OA should also be investigated in the future. Besides, OA of different sites were included. Studies are needed to determine if the benefits of n-3 PUFA supplementation for OA are consistent according to the sites of OA. Finally, long-term clinical studies with adequate sample size may be considered to explore the influence of n-3 PUFA supplementation on clinical outcomes in patients with OA.

## Conclusions

To sum up, results of the meta-analysis indicate that supplementation of n-3 PUFAs is effective to relieve pain and improve joint function in patients with OA, without increasing the risk of treatment related AEs. These findings support the use of n-3 PUFAs supplementation as an alternative treatment for OA.

## Data Availability

The present study was a meta-analysis of previous published studies.
